# PAYMENT POLICY IMPACT ON HOME HEALTH ACCESS FOR PEOPLE LIVING WITH DEMENTIA

**DOI:** 10.1093/geroni/igae098.0075

**Published:** 2024-12-31

**Authors:** Sara Knox, Daniel Brinton, Jada Johnson, Kit Simpson

**Affiliations:** Medical University of South Carolina, Charleston, South Carolina, United States; Medical University of South Carolina, Charleston, South Carolina, United States; Medical University of South Carolina, Charleston, South Carolina, United States; Medical University of South Carolina, Charleston, South Carolina, United States

## Abstract

Home health has been recommended as a strategy to prevent institutionalizations and promote aging in place for people living with dementia (PLWD). Longer home health episodes have been shown to optimize home health outcomes for PLWD. Recent payment policy reforms threaten the viability of this approach. The Patient-Driven Grouping Model (PDGM) uses patient characteristics to determine payment. Concerns have been raised that this model disincentivizes home health agencies from admitting patients, such as PLWD, who are admitted from the community and require longer episodes of care. We applied the PDGM to 100% home health files from 2019 to examine differences in PDGM components for PLWD and their peers before PDGM implementation. Two propensity score-matched groups of 153,957 individuals with and without dementia were created based on patient demographics, caregiver support, cognitive status, prior functional status, prior health care utilization, and Charlson comorbidity scores. Multivariate regression was used to examine the relationship between dementia and admission source/episode timing, functional limitations, and comorbidity level controlling for age, sex, and race. PLWD had greater odds of early community admissions (1.2 p<.0001), high functional limitation (1.09 p<.001), and of having a high comorbidity adjustment (1.49, p<.001). The primary clinical grouping for both groups was cardiac and circulatory. However, people with ADRD had twice as many Behavioral Health clinical group records. These findings indicate that people living with dementia did access and use home health differently before the implementation of the PDGM and may be experiencing negative repercussions of the new payment model.

